# Hybrid surgery of vertebral artery transposition combined with scallop and fenestration technique for the repair of type B aortic dissection patient with isolated left vertebral artery: A case report

**DOI:** 10.1097/MD.0000000000037410

**Published:** 2024-03-08

**Authors:** Shuxiong Ge, Zhongyou Xu, Jinlin Yan

**Affiliations:** a Department of Vascular Surgery, People’s Hospital affiliated to Ningbo University, Ningbo, Zhejiang.

**Keywords:** acute type B aortic dissection, fenestration, isolated left vertebral artery, landing zone, scallop, thoracic endovascular aortic repair, transposition

## Abstract

**Rationale:**

Acute type B aortic dissection (ABAD) is a fatal cardiovascular disease with high morbidity and mortality. Isolated left vertebral artery (ILVA) is a rare aortic arch mutation originating from the aortic arch. The simultaneous occurrence of both increases the complexity and difficulty of thoracic endovascular aortic repair. However, there have been few reports on the recommendation of thoracic endovascular aortic repair treatment strategies for aortic dissection patients concomitant ILVA with insufficient landing zone. Here, we report a case of ABAD combined with ILVA treated with hybrid surgery of left vertebral artery transposition alliance with Scallop and in vivo fenestration endograft.

**Patient concerns:**

A 38-year-old middle-aged man was transferred to our vascular department with persistent pain in his lower abdomen for 8 hours.

**Diagnoses:**

Preoperative computed tomography angiogram of the thoracic and abdominal aorta diagnosed with ABAD accompanied with ILVA.

**Interventions:**

Hybrid surgery of left vertebral artery transposition alliance with Scallop and in situ fenestration endograft for revascularization of ILVA, left subclavian artery, and left common carotid artery.

**Outcomes:**

The hybridization operation was successfully completed. There were no complications of cerebral and spinal cord ischemia after operation. Computed tomography angiogram examination indicated no internal leakage existed in the stent and patency of the arch vessels and the transposed left vertebral artery follow-up 3 months after surgery.

**Lessons:**

This study gave us experience in the treatment of aortic dissection with left vertebral artery variation and suggested that left vertebral artery transposition combined with scallop and in vivo fenestration stent is safe and effective.

## 1. Introduction

Acute type B aortic dissection is a rare and life-threatening cardiovascular disease with catastrophic consequences, occurring in approximately 3 cases per 100,000 per year.^[1]^ Aortic dissection (AD) refers to a tear in the intimal layer, allowing blood to enter the middle layer of the artery through the rupture in the intima, resulting in vulnerability. Most patients with AD are transferred to the emergency department due to chest and back pain resembling lacerations. The death rate for untreated patients is as high as 50%. With advancements in diagnosis and treatment technology, the diagnostic rate of AD is also increasing.^[2]^ Recently, thoracic endovascular aortic repair (TEVAR) has emerged as the recommended treatment strategy for AD.^[3]^ However, aortic arch revascularization poses challenges for TEVAR surgery due to the limited landing zone. Aortic arch vascular variation, although uncommon, is a significant concern as covering the arch vessels excessively can result in severe cerebral ischemic complications. Isolated left vertebral artery, as a form of aortic arch vascular malformation, presents a major challenge for aortic arch vascular reconstruction.^[4]^ Previously, due to surgical technique limitations, patients with type B AD combined with isolated left vertebral artery could only ensure blood flow to the brain through aortic arch vessel replacement, which had the disadvantage of causing significant trauma. In this study, our center successfully treated a patient with aortic B-type dissection and left vertebral artery variation using left vertebral artery transposition and the TEVAR technique.

## 2. Case report

**Figure 1.**
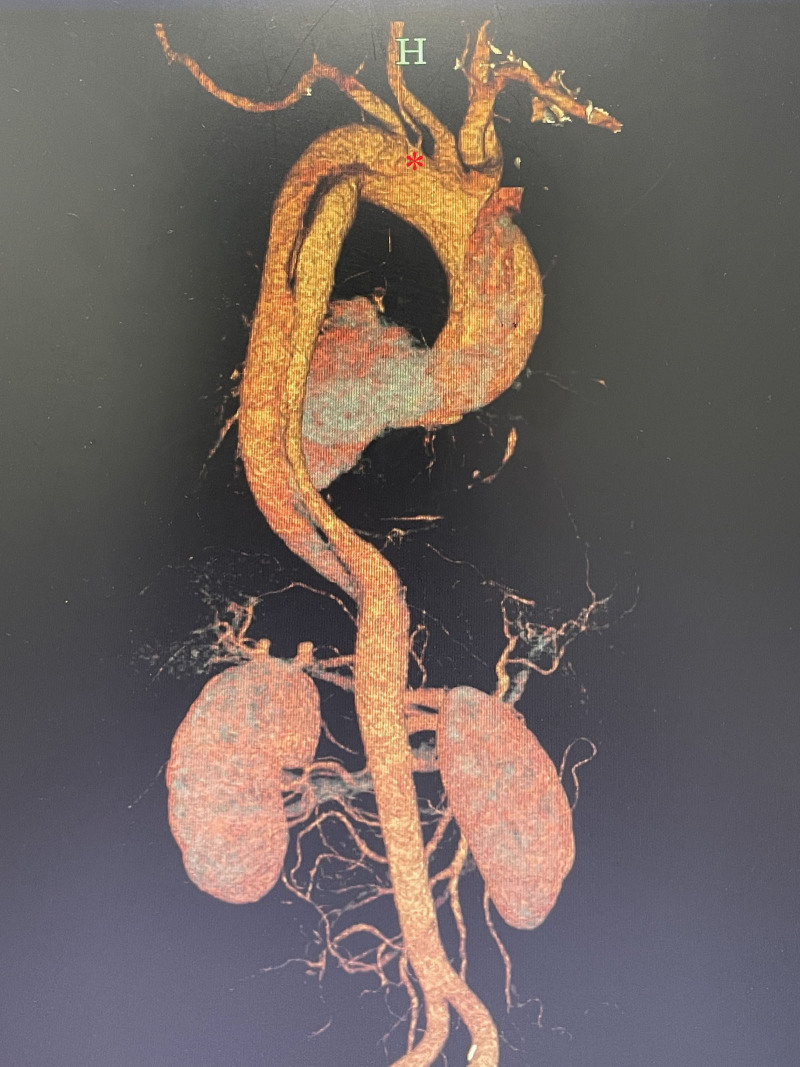
Preoperative CTA indicated that the aortic dissection and the left subclavian artery was derived from the aortic arch. *Isolated left vertebral artery. CTA = computed tomography angiogram.

A 38-year-old man was admitted to our vascular department complaining of persistent pain in his lower abdomen for 8 hours. The patient had a history of taking anti-hypertensive drugs and smoking. Emergency thoracic and abdominal aortic computed tomography angiography revealed an AD extending from the proximal left subclavian artery (LSA) to the abdominal trunk (Fig. 1). Surprisingly, the left vertebral artery originated from the aortic arch. After 2 weeks of hospitalization, the patient underwent stage I treatment, which involved left vertebral artery transposition combined with TEVAR technique.

**Figure 2.**
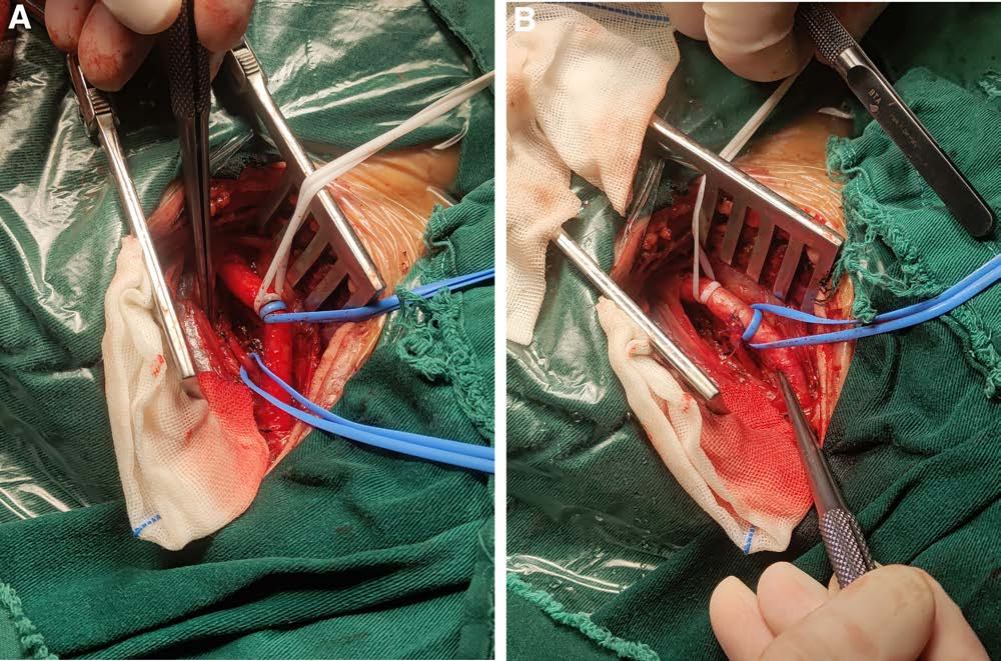
The procedure of left vertebral artery variation transposition. (A) The isolated left vertebral artery was separated behind the left common carotid artery. (B) The isolated left vertebral artery was transposed to the left common carotid artery.

Following successful general anesthesia, the patient was positioned supine and underwent an 8 cm longitudinal incision at the anterior edge of the left sternocleidomastoid muscle. The carotid sheath was then opened, allowing for complete dissociation of the common carotid artery, internal carotid artery, and external carotid artery. Careful identification of the nearby vagus nerve and internal jugular vein was also performed. The left vertebral artery, which runs behind the carotid artery, was exposed and fully separated (Fig. 2A). Systemic administration of 4000 units of heparin was then carried out, followed by blocking of the proximal and distal parts of the carotid arteries. The left vertebral artery was severed, with the aortic arch being partially covered and the distal part being anastomosed with the common carotid artery using an end-to-side anastomosis technique (Fig. 2B). The left neck incision was closed once the left vertebral artery resumed pulsation upon touch.

**Figure 3.**
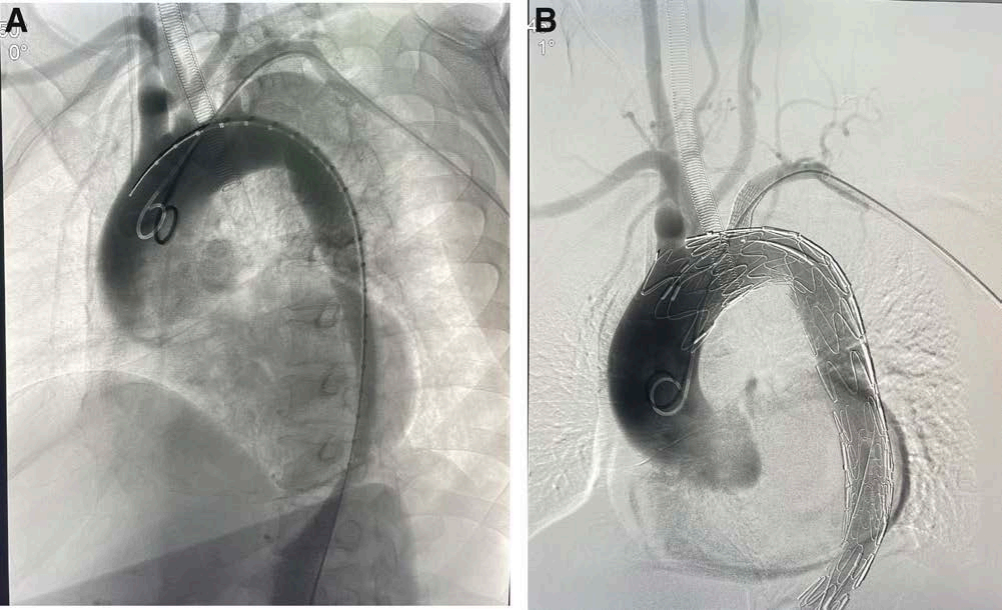
TEVAR reconstructed supraricular blood vessels using scallop and in situ fenestrating techniques. (A) DSA arteriography before revascularization. (B) DSA arteriography after revascularization. DSA = digital subtraction angiography, TEVAR = thoracic endovascular aortic repair.

**Figure 4.**
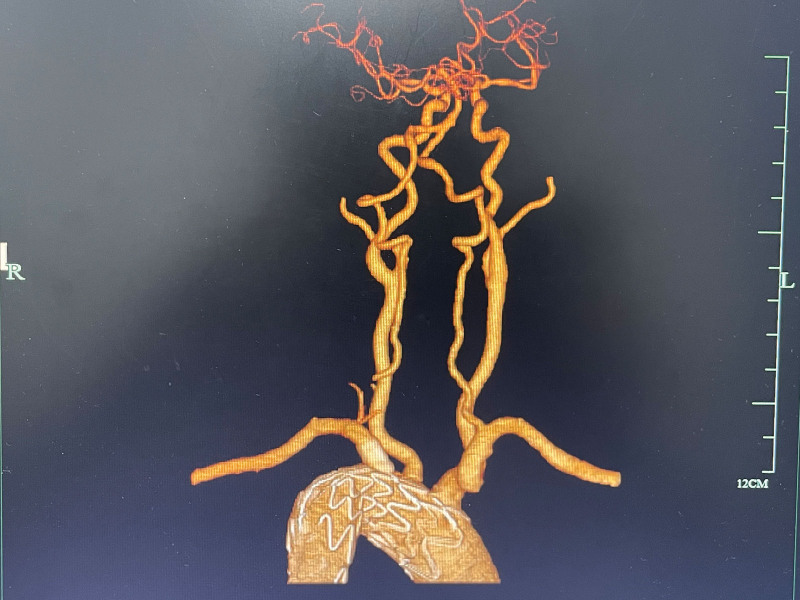
CTA indicated good postoperative outcome at 3 mo follow-up after hybridization. CTA = computed tomography angiogram.

Next, a 5-Fr sheath was inserted through the left brachial artery and right common femoral artery, respectively. The guide wire, combined with the catheter, successfully entered the ascending aorta through the true cavity accessed via the right femoral artery. Digital angiography revealed that the opening of the AD was located within 1.5 cm of the LSA, and the dissection involved the descending aorta (Fig. 3A). After replacing the superrigid guide wire, a 26 × 80 mm restricted cuff was placed at the level of the 11th thoracic vertebra. The main body support stent (40–32 mm × 200 mm Ankura, China) underwent physical modification with a 2 cm square scallop in vitro and a marker was sewed at the edge of the scallop. It was released when the distal marker reached the left common carotid artery. Then, the 6 F Fustar adjustable curved sheath (LifeTech Scientific Corporation, Shenzhen, China) and puncture needle were exchanged through the left brachial artery approach, and the proximal end of the 2 instruments was vertically placed against the membrane surface of the Ankura main stent. After performing multi-angle fluoroscopy and adjusting to the optimal position, the membrane was punctured and broken, and a 0.018 inch guide wire was introduced into the main stent. The balloon was then exchanged with diameters of 4, 6, and 8 mm to dilate the membrane. After adjusting the satisfaction angle, a 10 × 50 mm Viabahn stent was implanted under the guidance of roadmap, with the stent entering the aorta about 1 cm. Aortography revealed the perfect isolation of the dissection by the covered stent, as well as the patency of the brachiocephalic trunk, left common carotid artery, LSA, and transposed left vertebral artery (Fig. 3B). The right femoral artery was closed using a suture device, and the patient safely returned to the ward after recovering from general anesthesia. Computed tomography angiogram reexamination of the thoracic and abdominal aorta 3 months after surgery showed no internal leakage of the aortic coated stent, and confirmed the patency of the left common carotid artery, LSA, and transposed left vertebral artery (Fig. 4). This study was approved by the Ethics Committee of the Affiliated People’s Hospital of Ningbo University.

## 3. Discussion

AD has now entered the era of total endovascular treatment, with TEVAR being the preferred procedure for treating TBAD. This therapy has shown significant reduction in mortality rates and improved prognosis for patients with type B AD.^[5]^ However, the conventional TEVAR treatment is limited in cases where the lesion involves partial branches of the aortic arch, particularly the LSA, due to the lack of a proximal anchor area for the stent. In order to successfully treat Stanford Type B aortic dissection, it is crucial to effectively seal the dissection rupture and ensure blood supply to the important branches of the aortic arch. The vertebral artery, which branches off from the subclavian artery, must be preserved during TEVAR treatment of AD to ensure proper blood supply to the posterior cerebral circulation. Apart from the challenge of inadequate anchoring areas, the variation in aortic arch vasculature poses another obstacle for TEVAR.

The normal vertebral artery, originating from the subclavicular artery, joins the basilar artery to form the circle of Willis. It serves as a crucial source of blood supply to the brainstem, cerebellum, and the posterior hemispheres of both cerebral hemispheres. However, there are anatomical variations in the aortic arch, and the most common variation is the left vertebral artery directly originating from the aortic arch (2.8%–4.2%).^[6]^ Consequently, the presence of left vertebral artery variation significantly impacts the endovascular treatment strategy of AD.^[7]^

Arch reconstruction, preserving all the supra-arch vessels, is the standard treatment for patients with arch anomalies. Currently, there are several common methods used to reconstruct the supra-arch vessels with isolated left vertebral artery. Previous surgical treatments, which aimed to preserve all the blood vessels in the arch through aortic arch replacement, have been phased out due to high mortality and trauma.^[8]^ With the advancement of endovascular therapy, the chimney technique and fenestration technique have become important in preserving the left vertebral artery. However, internal leakage and stent structure damage are inevitable drawbacks.^[9]^ Transposition from the left vertebral artery to the left carotid artery or graft bypass is commonly used to manage vertebral artery variation. This technique has a high success and patency rate, with fewer complications.^[10]^

The proximal landing zone’s appropriate length is crucial for TEVAR surgery in patients with AD as it contributes to the long-term durability of stent implantation. However, factors such as the angle of aortic arch angulation, the distance of branch vessels in the aortic arch, and the short neck length due to reverse aortic involvement can interfere with the suitability of the proximal landing zone.^[11]^ To ensure sufficient anchoring area, the stent needs to be extended forward, which may inadvertently cover the major supraricular vessels and affect blood supply to the brain. Currently, a common approach is to reconstruct the suprachial blood vessels through fenestration and branch stent techniques, which have shown a certain success rate and patency rate. Fernández-Alonso et al^[12]^ reported that scalloped or fenestrated manual modification grafts offer an applicable technique for managing patients with aortic arch disease. Lucien et al^[13]^ demonstrated that scalloped or fenestrated stents, physician-modified for the reconstruction of the LSA, are considered safe and durable in the midterm. A previous study reported successful treatment of penetrating aorta ulcer with an isolated left vertebral artery through in situ fenestration and chimney technique for revascularization of isolated left vertebral artery and the LSA.^[14]^ In order to obtain sufficient anchoring area, we created a scallop at the beginning of the coated stent to extend it to the middle of the left common carotid artery. Additionally, we used the in situ fenestration technique to reconstruct the LSA and transposed the left vertebral artery to the left carotid artery to avoid stent coverage in this patient with AD. Importantly, there were no complications of cerebral and spinal cord ischemia after the operation. Computed tomography angiogram examination revealed no internal leakage in the stent and confirmed the patency of the arch vessels, as well as the transposed left vertebral artery, during the 3-month follow-up after surgery. These findings demonstrate the safety and feasibility of the modified hybrid surgery.

## 4. Conclusion

The alliance between vertebral artery transposition, Scallop, and in situ fenestration endograft ensures that the landing zone is sufficiently long to support the stability and sealing of the stent. This innovative hybrid surgery technique has demonstrated both safety and effectiveness in patients with AD and an aberrant left vertebral artery.

## Author contributions

**Software:** Shuxiong Ge.

**Writing – original draft:** Shuxiong Ge.

**Supervision:** Zhongyou Xu, Jinlin Yan.

**Writing – review & editing:** Zhongyou Xu, Jinlin Yan.
